# Modeling Current and Future Habitat Suitability for the Snow Leopard (*Panthera uncia*) Under Climate Change Scenarios in Nepal

**DOI:** 10.1002/ece3.72490

**Published:** 2025-11-12

**Authors:** Meghajan Budha, Jharana Karki, Barsha Khadka, Narayan Prasad Koju

**Affiliations:** ^1^ Department of Environmental Science GoldenGate International College, Tribhuvan University Kathmandu Nepal; ^2^ Center for Post Graduate Studies Nepal Engineering College, Pokhara University Bhaktapur Nepal; ^3^ Department of Psychology University of Washington Seattle Washington USA; ^4^ Key Laboratory of Genetic Evolution and Animal Models, Kunming Institute of Zoology, Chinese Academy of Sciences Kunming Yunnan China

**Keywords:** climate resilience, environmental variables, Himalayas, protected areas, species distribution modeling, SSP scenarios

## Abstract

The snow leopard ( *Panthera uncia* ), a Vulnerable apex predator endemic to the mountainous regions of Central and South Asia. It plays a vital role in maintaining the ecological integrity of high‐altitude ecosystems. This study modeled the current and future potential habitat distribution of the snow leopard in Nepal using Species Distribution Modeling (SDM). A total of 306 occurrence records were compiled from both primary and secondary sources. Five bioclimatic and four environmental variables were selected to assess their influence on habitat suitability, and the MaxEnt algorithm was used to develop distribution models. Results indicate that nearly one fifth of Nepal's total land area is suitable for snow leopards. Most of these suitable habitats lie within the protected areas (PAs). However, a significant portion of suitable habitat in the western landscapes extends into vulnerable, unprotected regions. Among the environmental variables, annual mean temperature and elevation emerged as the most influential predictors. Habitat suitability was highest in areas with lower temperatures (−5°C to 5°C) and within the elevation range of 4000–4500 masl. Climate projections for mid and late century highlight a substantial concentration of moderately and marginally suitable habitats, with particular severe declines under high emission scenarios. While protected areas were found to provide relatively resilient habitats for the snow leopard, areas outside the PAs network are projected to undergo significant habitat contraction. This emphasizes the urgent need for expanded and adaptive conservation strategies. Notably, this study is the first to quantify the disproportionate vulnerability of habitats outside Nepal's protected area system. In the western region, approximately 42.5% of currently suitable habitat is at risk of severe decline under high‐emission scenarios. These findings highlight the limitations of existing conservation paradigms and emphasize the need to extend protections beyond established PAs through the creation of ecological corridors and the integration of climate‐resilient conservation planning.

## Graphical abstract

This study models current and future snow leopard habitat in Nepal, revealing that 19.41% of the country is suitable, with 42.53% outside protected areas. Under high‐emission scenarios, significant habitat loss is projected, especially outside PAs, underscoring the need for expanded, climate‐resilient conservation strategies.

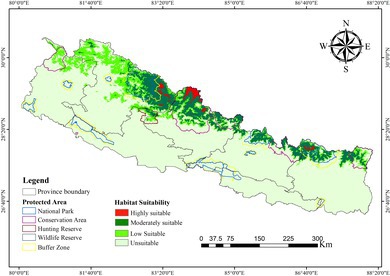

## 1 Introduction

The snow leopard ( *Panthera uncia* ), a large felid species native to the mountainous regions of Central and South Asia, is currently classified as “Vulnerable” by the International Union for Conservation of Nature (IUCN) due to declining population trends (Sanyal et al. 2024). As an apex predator, the snow leopard plays a vital ecological role by regulating herbivore populations and maintaining the stability of alpine ecosystems (Oberosler et al. 2022). Its morphological adaptations, including dense fur, a long tail, shortened limbs, and enlarged nasal cavities, enable it to thrive in high‐altitude environments (Kazmi et al. 2021).

Snow leopards primarily prey on Siberian ibex, blue sheep, Himalayan Tahr, Himalayan musk deer, marmots, and pikas underscoring their dependence on healthy herbivore populations for survival (Hacker 2021; Lyngdoh et al. 2014; Sanyal et al. 2024) and also exhibit seasonal plasticity in diet (Koju et al. 2023). However, this species faces increasing threats from habitat loss due to climate change and competition with other predators like the leopard (Buzzard et al. 2017; Kazmi et al. 2021; Koju et al. 2024; Lovari et al. 2024, 2013) and from anthropogenic pressures including poaching and retaliatory killings (Filla et al. 2022; Ikeda 2004; Moheb et al. 2023).

The global distribution of the snow leopard spans across 12 countries in Central and South Asia, typically at elevations between 3000 and 5500 m in arid and semi‐arid shrub‐lands, grasslands, and steppes (Chen et al. 2017; Feng et al. 2022; Koju et al. 2021; Mahmood et al. 2019; McCarthy et al. 2024). After being listed as “Endangered” for 45 years, the snow leopard's IUCN status was reclassified to “Vulnerable” in 2017, although the species remains listed in appendix I of the Convention on International Trade in Endangered Species (CITES) (McCarthy et al. 2017). Spatial analysis suggests a historical range contraction of approximately 69%, reinforcing the urgent need for strategic conservation interventions (Li et al. 2020; Mahmood et al. 2019). The continued threats from illegal wildlife trade, including the demand for pelts, bones, and body parts for traditional medicine, further endanger the species (Lyngdoh et al. 2014; Nowell 2016; Theile 2003).

Nepal is home to an estimated 397 snow leopards (approximately 12% global population), primarily distributed in the northern Himalayan region along the borders with China and India (DNPWC and DoFSC 2025). According to GSLEP (2013), Nepal has the second smallest snow leopard habitat, yet it hosts the fourth largest population in the world. The snow leopard habitat in Nepal is divided into three conservation landscapes: Eastern, Central and Western (DNPWC and DoFSC 2024). Key protected areas within Nepal's western conservation landscapes that document the presence of the snow leopard include Api‐Nampa Conservation Area, Dhorpatan Hunting Reserve, and Shey Phoksundo National Park. In central landscapes, snow leopards have been recorded in Manaslu Conservation Area and Annapurna Conservation Area. Similarly, in eastern conservation landscapes, key sites include Gaurishankar Conservation Area, Langtang National Park, Kanchenjunga Conservation Area, Makalu Barun National Park, and Sagarmatha National Park (Aryal et al. 2010; DNPWC and DoFSC 2025; Jnawali et al. 2011; Koju et al. 2024).

Beyond the protected areas network, snow leopards have been reported from several regions, including the eastern part of Dolpa (Chharka Tangsong, Kaike, and Dolpo Buddha Rural Municipality) and the Limi Valley of Humla district (Lama et al. 2018; WWF 2024). Notable valleys supporting snow leopard populations include Lapchi and Rolwaling Valley in Gaurishankar Conservation Area, Limi Valley in Humla district, Nar Phu Valley in Annapurna Conservation Area, and Tshum Valley in Manaslu Conservation Area (DNPWC and DoFSC 2024, 2025; Koju et al. 2024, 2023; Lama et al. 2018; Thapa and Rayamajhi 2023). However, existing studies remain limited, especially concerning the species' ecological responses to environmental stressors, including climate change (McCarthy et al. 2024).

Domesticated alpine herbivores that share habitats with snow leopards include yaks, goats, horses, and sheep (Chetri et al. 2017; Karki and Panthi 2021; Koju et al. 2024, 2023; Tiwari et al. 2020). Predator–prey interactions shape the behavior of both groups and influence overall ecosystem integrity (Estes et al. 2011; Piquet et al. 2018). Among anthropogenic disturbances, livestock grazing is a major driver of wild ungulate population declines and alters the predator–prey dynamics (Xu et al. 2024). The intensity of livestock depredation largely depends on the snow leopard's abundance and livestock availability, though depredation rates may vary irrespective of the wild prey density level (Khanal et al. 2020). Livestock depredation by the snow leopard is widespread across its range (Jackson et al. 2010). Chetri et al. (2017) reported that the preference of snow leopards is horses and goats, whereas Tiwari et al. (2020) and Koju et al. (2023) found that most depredation incidents involving the snow leopard are yaks, followed by goats, sheep, and horses. In Nepal, snow leopard depredation causes substantial annual livestock losses, resulting in significant economic impacts on local communities and fueling human–snow leopard conflict, a major challenge in the conservation of the snow leopard (Karki and Panthi 2021).

Species distribution models (SDMs) are widely used in ecology and conservation to interpolate and extrapolate species distributions using quantitative or rule‐based approaches (Zurell et al. 2020). They help to predict occurrence based on environmental conditions, serving as proxies for habitat suitability (Warren and Seifert 2011). SDMs are applied to describe species distributions, characterize species‐habitat relationships, and predict species responses to environmental change, thereby informing management and conservation decision‐making (Zurell et al. 2022). The increasing availability of large ecological datasets and the complexity of species‐environment interactions have expanded the use of SDMs, including ecological niche modeling, habitat modeling, predictive habitat distribution modeling, and range mapping (Beery et al. 2021). Importantly, SDMs also provide critical insights into the potential effects of climate change on species distribution, a cornerstone of climate‐resilient conservation planning (Forester et al. 2013; Raymond et al. 2020).

This study addresses critical knowledge gaps by analyzing the current spatial distribution of snow leopards in Nepal and projecting future habitat shifts under multiple climate change scenarios. These findings aim to inform evidence‐based conservation strategies, particularly in western Nepal, where severe habitat losses are projected outside the protected area.

## 2 Materials and Methods

### 2.1 Study Area

**FIGURE 1:**
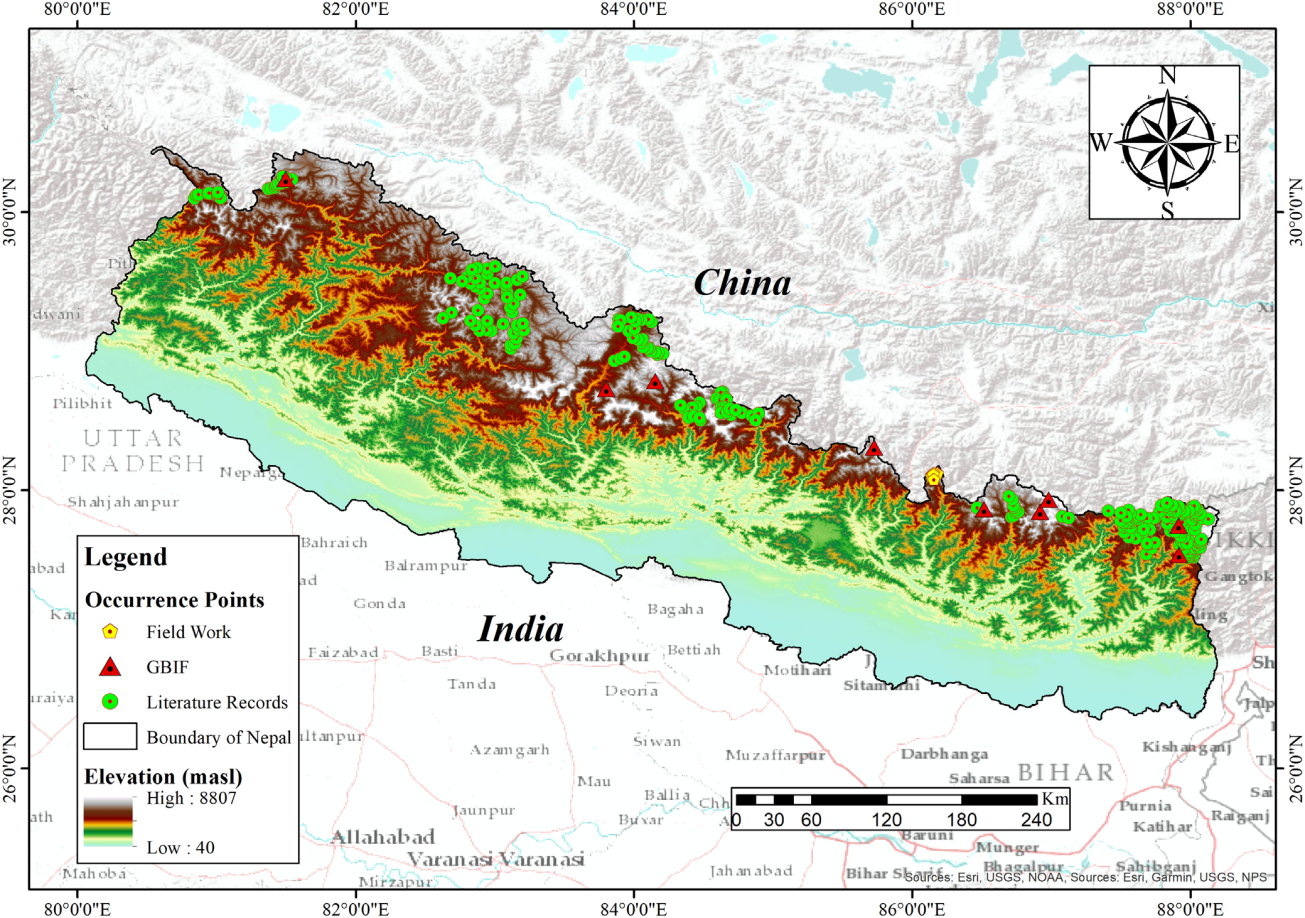
Map of the study area showing occurrence points of snow leopards.

Nepal, a landlocked country in South Asia, spans an area of 147,516 km^2^. It is bordered by China to the north and India to the east, south, and west (Bhattacharjee et al. 2017; Dhami et al. 2024; Rai et al. 2001). Geographically, the country spans about 885 km from west to east, lying between longitudes 80°04′ and 88°12′ east and latitudes 26°22′ and 30°27′ north (Figure 1) (Baral 1986; Dhami et al. 2024). Nepal exhibits extreme topographical variation, ranging from the lowland Terai at ~60 m to the peak of Mount Everest at 8848.86 m. This elevational gradient supports a remarkable diversity of ecosystems and climatic zones, spanning from tropical to nival (Bhattacharjee et al. 2017; Gurung et al. 2024).

The country is broadly divided into three physiographic regions: lowland Terai plains, mid‐hills, and higher Himalayas (Shrestha and Aryal 2011). Nepal receives substantial rainfall, particularly during the monsoon season, with an average annual precipitation of 1768 mm (Gurung et al. 2024; Shrestha et al. 2000). Temperatures vary widely, from hot and humid conditions in the Terai to freezing in the high Himalayas with an annual mean temperature of 18°C (Shrestha et al. 2000). About 86% of Nepal's terrain comprises hills and mountains, providing suitable ecological niches for snow leopards and their prey species (Aryal et al. 2016; Bhuju et al. 2007).

Nepal is one of the key protected regions within the Global Snow Leopard and Ecosystem Protection Program (GSLEP), encompassing three of the 24 priority landscapes for snow leopard conservation (DNPWC 2017; DNPWC and DoFSC 2025). Out of the three conservation landscapes in Nepal, the highest densities are reported from the western landscape, followed by the central and eastern landscapes (DNPWC and DoFSC 2025).

### 2.2 Data Collection

#### 2.2.1 Snow Leopard Occurrence Data

Snow leopard occurrence records were compiled from both primary and secondary sources. Primary data were collected from the Lapchi Valley of Gaurishankar Conservation Area through sign survey and systematic camera trap deployment. The study area was divided into 69 grid cells, each measuring 2 × 2 km. A total of 26 camera traps were strategically installed between 2018 and 2024, based on terrain features, vegetation types, and the presence of scats, scrapes, or prey signs (Koju et al. 2024).

Additionally, scat samples (collected for a diet analysis study, manuscript under review) were genetically analyzed to confirm snow leopard identity. DNA was extracted and amplified using cytb‐targeted primers (CYTB‐SCT‐PUN‐F/R) developed by Janečka et al. (2008). During PCR, a positive control of a reference snow leopard DNA confirmed by d‐loop sequencing was used. Only genetically confirmed snow leopard scat sample data were retained for the analysis.

From field surveys, 25 snow leopard presence locations were documented, comprising 9 from camera traps and 16 from sign surveys. Additionally, 281 snow leopard occurrence records were obtained from the Global Biodiversity Information Facility (GBIF) (https://www.gbif.org/), setting the cut‐off date at 2000 AD, and peer‐reviewed literature (Islam et al. 2023; Watts et al. 2019). A total of 306 unique occurrence records were available for analysis. Secondary sources included GBIF (2025), Ale et al. (2007), Byers et al. (2014), DNPWC and DoFSC (2024), DNPWC and DoFSC (2025), Karki and Panthi (2021), Lama et al. (2018), Pandey et al. (2021), Thapa et al. (2021), Timilsina et al. (2024), Upadhyay (2010) and ANCA (2018).

#### 2.2.2 Environmental Variables

Environmental predictors were compiled to model the habitat suitability of the snow leopard. Data on 19 bioclimatic variables (~1 km resolution) were obtained from the WorldClim database (Fick and Hijmans 2017) (https://www.worldclim.org/, accessed on 16th February, 2025). Land cover data were obtained from the Esri Land Cover (https://livingatlas.arcgis.com/landcover/, accessed on 15th February, 2025). Soil type data were accessed from the FAO Soil Portal (https://www.fao.org/, accessed on 17th February, 2025). Elevation data from the Shuttle Radar Topography Mission (SRTM) (https://earthexplorer.usgs.gov/, accessed on 12th February, 2025) were used to derive slope and aspect using ArcGIS tools.

Recently, the Intergovernmental Panel on Climate Change (IPCC) published its sixth assessment report (AR6) (IPCC 2023), which introduced a Coupled Model Intercomparison Project Phase 6 (CMIP6). According to IPCC (2023), CMIP6 models demonstrate significant advancements in both qualitative and quantitative dimensions compared to their previous phases, such as CMIP3 and CMIP5. So, for future projections, data having a resolution of 30 arc‐seconds (~1 km) were derived from the BCC‐CSM2‐MR model under this CMIP6 framework. Four Shared Socio‐economic Pathways (SPPs) were used: SSP1‐2.6 (low emissions), SSP2‐4.5 (intermediate emissions), SSP3‐7.0 (high emissions), and SSP5‐8.5 (very high emissions). Projections were made for the mid‐century (2041–2060) and late‐century (2061–2080) periods (Chen et al. 2022; Harris et al. 2023; Luo et al. 2025). All environmental layers were resampled to a uniform spatial resolution of 30 arc‐seconds (~1 km) using the “resample” tool in ArcGIS, ensuring consistency with bioclimatic variables, and the projection system of all the variables was made uniform using the “project raster” tool in ArcGIS (Islam et al. 2023) (Table 1).

**TABLE 1:** Details of Environmental variables with their sources.

Abbreviation	Environmental variables	Original resolution	Sources
Bio 1	Annual Mean Temperature	30 arc‐seconds (~1 km)	https://www.worldclim.org/
Bio 2	Mean Diurnal Range (Mean of monthly (max temp–min temp))		
Bio 3	Isothermality (BIO2/BIO7) (×100)		
Bio 4	Temperature Seasonality (standard deviation ×100)		
Bio 5	Max Temperature of Warmest Month		
Bio 6	Min Temperature of Coldest Month		
Bio 7	Temperature Annual Range (BIO5–BIO6)		
Bio 8	Mean Temperature of Wettest Quarter		
Bio 9	Mean Temperature of Driest Quarter		
Bio 10	Mean Temperature of Warmest Quarter		
Bio 11	Mean Temperature of Coldest Quarter		
Bio 12	Annual Precipitation		
Bio 13	Precipitation of Wettest Month		
Bio 14	Precipitation of Driest Month		
Bio 15	Precipitation Seasonality (Coefficient of Variation)		
Bio 16	Precipitation of Wettest Quarter		
Bio 17	Precipitation of Driest Quarter		
Bio 18	Precipitation of Warmest Quarter		
Bio 19	Precipitation of Coldest Quarter		
Slope	Slope	30 m	https://earthexplorer.usgs.gov/
Aspect	Aspect	30 m	
Elev	Elevation	30 m	
Soil	Soil Type	—	https://www.fao.org/
Land cover	Land Cover Type	10 m	https://livingatlas.arcgis.com/landcover/

### 2.3 Data Analysis

#### 2.3.1 Spatial Filtering and Variable Selection

Spatial filtering was applied to reduce autocorrelation and sampling bias in snow leopard presence records. To improve the quality of presence location data for model calibration and evaluation, autocorrelated points were filtered from the presence dataset of the snow leopard (Aryal et al. 2016; Boria et al. 2014; Radosavljevic and Anderson 2014; Watts et al. 2019). This procedure was performed using the “SDM” toolbox, a Python‐based GIS toolkit in ArcGIS, retaining only one record per 5 km grid cell (Aryal et al. 2016; Brown 2014). After spatial filtering, 127 unique occurrence points were used for the modeling.

**FIGURE 2:**
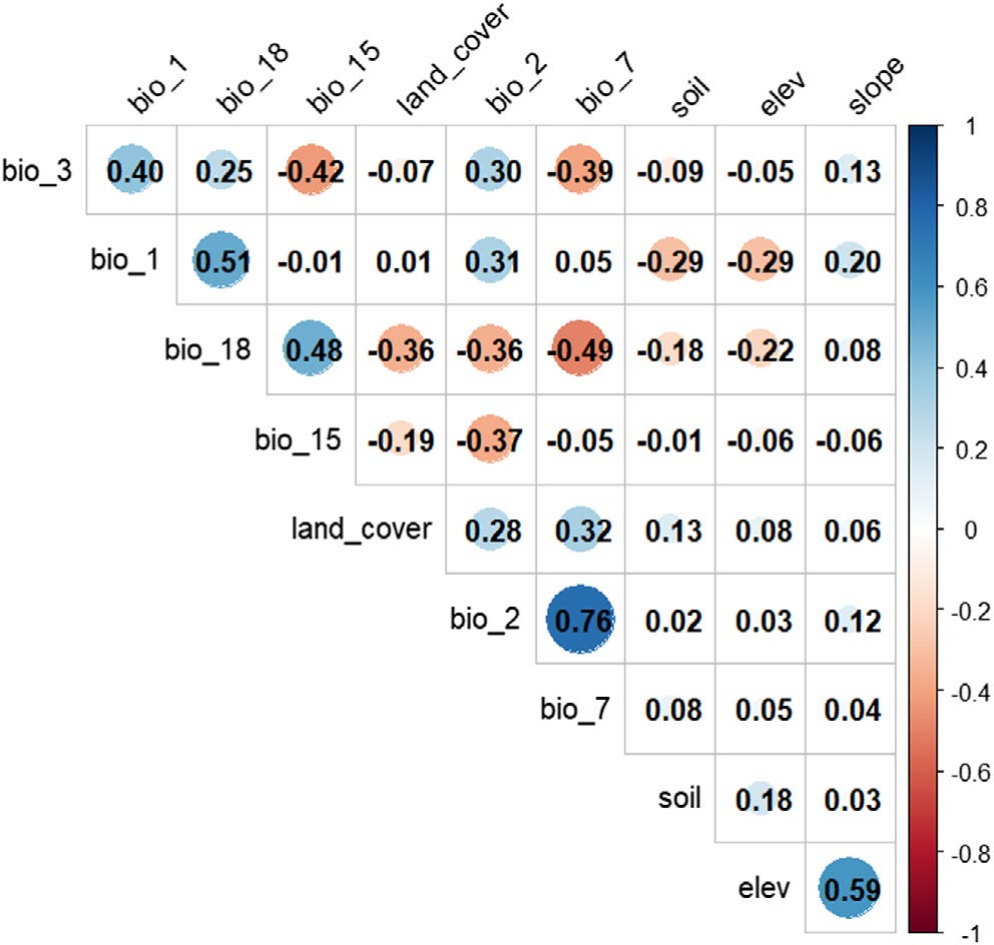
Correlation coefficient of bioclimatic and environmental variables used in the modeling.

To address multicollinearity among environmental variables, the Pearson correlation coefficient (Figure 2) was calculated using the “corrplot” package in RStudio (Wei et al. 2024). Variables with correlation coefficients greater than 0.80 were excluded (Aryal et al. 2016; Ismaili et al. 2024). Based on ecological relevance and low multicollinearity, five bioclimatic variables (Bio1, Bio2, Bio7, Bio15, and Bio18) and four environmental variables (land cover type, soil type, slope, and elevation) were selected for the modeling (Aryal et al. 2016; Islam et al. 2023; Ismaili et al. 2024; Rashid et al. 2021).

Climatic variables were prioritized, with annual mean temperature (Bio1) considered the most critical factor, as snow leopards are adapted to cold alpine zones and are threatened by rising temperatures affecting both the species and its prey (Kazmi et al. 2021; MoFSC 2017). Mean diurnal range (Bio2), temperature annual range (Bio7), and precipitation seasonality (Bio15) were included due to their influence on physiological stress, vegetation patterns, and prey availability through seasonal dynamics (Jianhui et al. 2023; Kazmi et al. 2021).

Topographic variables of slope and elevation were incorporated as snow leopards prefer rugged slopes around 24°–25° and elevations of 3000–5000 m, which provide hunting cover and regulate temperature regimes critical for prey availability (Bai et al. 2018; Lham et al. 2021; MoFSC 2017; Shrestha and Kindlmann 2020). Land cover and soil type were included to capture habitat structure effects, as snow leopards associate with rocky alpine meadows and sparse vegetation above the tree line, which influences prey distribution, denning sites, and the broader ecosystem that supports key prey species (Bai et al. 2018; Ismaili et al. 2024).

#### 2.3.2 Maximum Entropy Model Parameter Setting

MaxEnt Version 3.4.4 (https://biodiversityinformatics.amnh.org/open_source/maxent/) (Phillips et al. 2006) was used to model the habitat suitability of the snow leopard in Nepal. All the selected bioclimatic variables and environmental variables were first converted into ASCII (American Standard Code for Information Interchange) format in ArcGIS. The variables and snow leopard occurrence data were then imported into MaxEnt. The model was trained using a random 75% subset of the presence records, with the remaining 25% used for testing, employing the bootstrap method with ten replications, while other settings were kept at default (Aryal et al. 2016; Islam et al. 2023; Jianhui et al. 2023; Watts et al. 2019).

Model accuracy was evaluated using the receiver operator characteristic (ROC) curve (Ismaili et al. 2024; Jianhui et al. 2023) and true skill statistics (TSS), as recent studies suggest that AUC was insufficient for assessing spatial distribution model performance (Li et al. 2023; Lobo et al. 2008). Following the study by Liu et al. (2016), the maximum sum of sensitivity and specificity (maxSSS) threshold was selected, and TSS average from ten repeated MaxEnt runs was calculated using the “Presence‐Absence” package in RStudio (Li et al. 2023). Models with an AUC value less than 0.70 were considered poor, 0.7–0.9 moderate, and 0.9–1.0 good (Hajian‐Tilaki 2013; Wang et al. 2021). The TSS values of 0.2–0.5 indicate poor performance, 0.6–0.8 useful model performance, and > 0.8 excellent performance (Gama et al. 2017; Li et al. 2023).

Additionally, the jackknife method of regularized training gain was applied to assess the individual contribution of each variable. This analysis compares the training gain of each variable in isolation with the training gain when all variables are included, identifying the most influential predictors (Phillips et al. 2006; Young et al. 2011).

#### 2.3.3 Habitat Suitability Classification System

The average ASCII file was imported into ArcGIS, and the projection of the file was defined using the “define projection” tool. The file was then converted into the floating‐point raster data using the “Raster calculator” tool (Jianhui et al. 2023). To classify habitat suitability for the snow leopard, the maximum training sensitivity and specificity logistic threshold value (0.3463) was used to differentiate suitable and unsuitable areas (Jianhui et al. 2023; Liu et al. 2005; Zahoor et al. 2021). The habitat suitability was further divided into four classes: 0–0.3463 as unsuitable area, 0.3463–0.50 as low suitable area, 0.50–0.70 as moderately suitable area, and 0.70–1.0 as highly suitable area (Baral et al. 2023). The area of each class was calculated by multiplying the number of cells by the area of a cell using the “Raster calculator” tool in ArcGIS. Through this, the snow leopard habitat suitability distribution across Nepal was determined.

## 3 Results

### 3.1 Habitat Suitability Mapping

**FIGURE 3:**
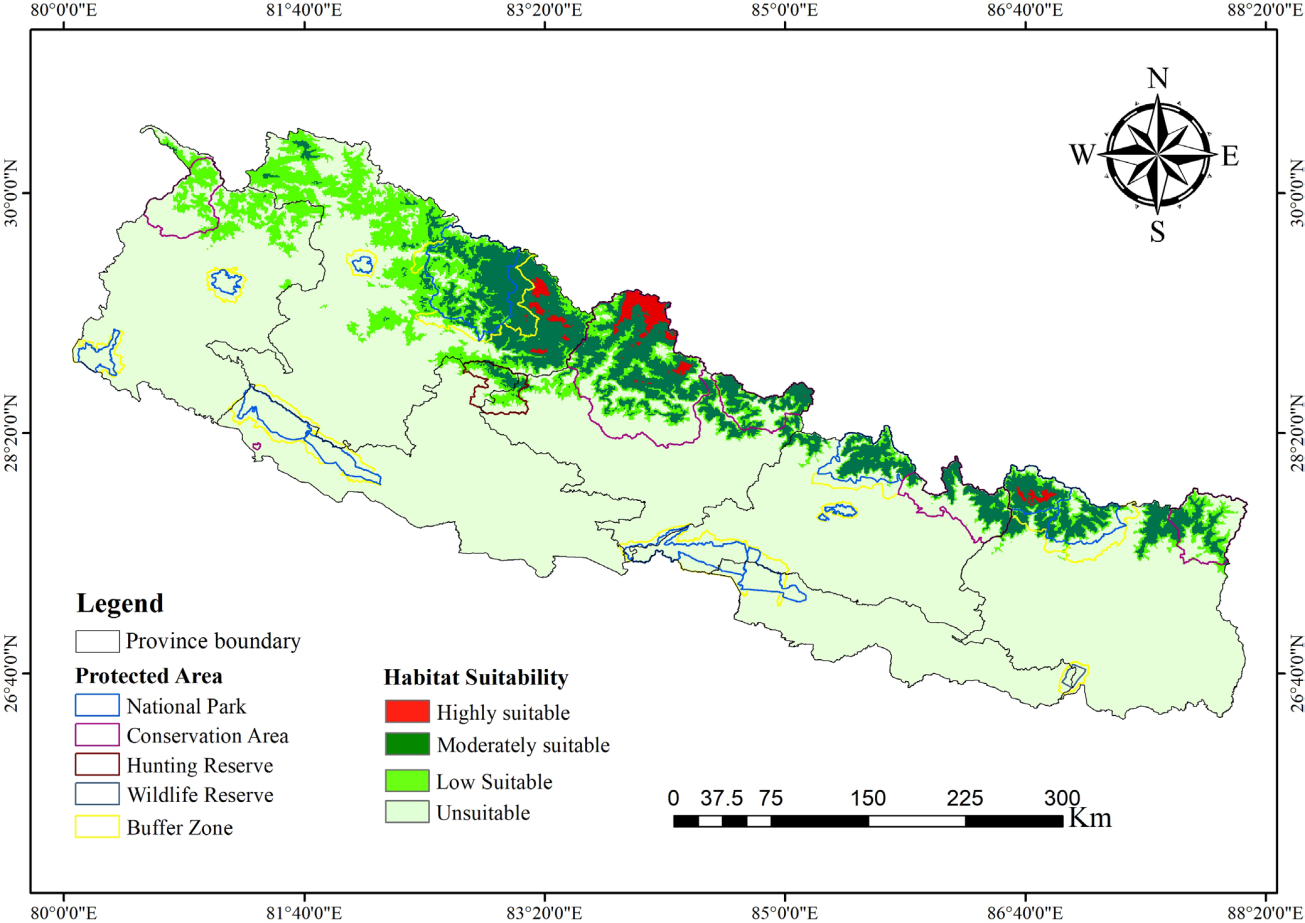
Habitat Suitability Map of snow leopards in Nepal.

Spatial modeling of suitable habitat for the snow leopard across Nepal identified approximately 19.41% (28,626.81 km^2^) of the country as potentially suitable for the species (Figure 3). Of the total suitable habitat identified, 57.47% (16,451.27 km^2^) lies within protected areas, while the remaining 42.53% (12,175.54 km^2^) lies outside the formal PAs. Karnali Province harbors the largest extent of suitable habitat (12,176.92 km^2^), whereas Madhesh and Lumbini Provinces have the least, with 0 km^2^ and 451.79 km^2^, respectively (Table 2). Among protected areas, the Annapurna Conservation Area encompasses the largest suitable habitat (5019.01 km^2^), followed by Shey‐Phoksundo National Park and its buffer zone (3968.29 km^2^). The Dhorpatan Hunting Reserve contains the smallest suitable area (545.48 km^2^) (Table 3).

**TABLE 2:** Province‐wise distribution of snow leopard habitat suitability areas outside protected areas in Nepal.

Name of province	Highly suitable		Moderately suitable		Low suitable		Total		Total suitable area outside PA	
	Area (Km^2^)	%	Area (Km^2^)	%	Area (Km^2^)	%	Area (Km^2^)	%	Area (Km^2^)	%
Koshi	113.82	11.29	2494.24	18.44	1486.55	10.55	4094.60	14.30	955.64	7.85
Bagmati	0.69	0.07	1696.14	12.54	602.39	4.28	2299.22	8.03	281.07	2.31
Gandaki	635.70	63.09	4236.18	31.31	2614.99	18.56	7486.87	26.15	1154.12	9.48
Karnali	257.47	25.55	4932.95	36.46	6986.50	49.58	12,176.92	42.54	8208.63	67.42
Sudurpaschim	0.00	0.00	11.80	0.09	2105.60	14.94	2117.39	7.40	1496.26	12.29
Lumbini	0.00	0.00	156.84	1.16	294.95	2.09	451.79	1.58	79.81	0.66
Madhesh	0.00	0.00	0.00	0.00	0.00	0.00	0.00	0.00	0.00	0.00
Total	1007.69	100.00	13,528.14	100.00	14090.98	100.00	28626.81	100.00	12,175.54	100.00

**TABLE 3:** Suitability area of *Panthera uncia* within the protected area of Nepal.

Name of PA	Total area of PA	Highly suitable (Km^2^)	Moderately suitable (Km^2^)	Low suitable (Km^2^)	Total suitable area (Km^2^)	Suitable area percentage (%)
Kanchenjunga Conservation Area	2035	0.00	512.17	548.95	1061.13	6.45
Makalu Barun National Park	1500	0.00	574.63	310.22	884.85	5.38
Makalu Barun Buffer Zone	830	0.00	14.57	45.11	59.68	0.36
Sagarmatha National Park	1148	113.82	594.06	180.44	888.32	5.40
Sagarmatha Buffer Zone	275	0.00	179.75	65.24	244.98	1.49
Gaurishankar Conservation Area	2179	0.69	725.23	217.92	943.84	5.74
Langtang National Park	1710	0.00	800.18	268.58	1068.76	6.50
Langtang Buffer Zone	420	0.00	0.00	5.55	5.55	0.03
Manaslu Conservation Area	1663	0.69	793.94	345.61	1140.24	6.93
Annapurna Conservation Area	7629	634.32	2867.61	1517.08	5019.01	30.51
Dhorpatan Hunting Reserve	1325	0.00	158.93	386.56	545.48	3.32
Shey‐Phoksundo National Park	3555	0.00	1753.04	1220.05	2973.10	18.07
Shey‐Phoksundo Buffer Zone	1349	4.86	718.29	272.05	995.20	6.05
Api Nampa Conservation Area	1903	0.00	0.00	621.13	621.13	3.78
		754.38	9692.40	6004.49	16,451.27	100.00

### 3.2 Spatial Heterogeneity in Habitat Suitability

The spatial analysis revealed marked heterogeneity in habitat suitability across Nepal's conservation landscapes. The western landscape (mainly Karnali and Sudurpaschim Provinces) harbors the largest extent of suitable habitat (53.19% of the total). The central landscape comprises approximately 25.45% of the area. The eastern landscape represents the remaining 21.36%. Highly suitable zones are spatially clustered around the Shey‐Phoksundo and Annapurna regions. Moderate and low suitability zones are more dispersed in the central Himalayas.

### 3.3 Environmental Variable Contributions and Model Performance

The MaxEnt model was used to predict the potential distribution of snow leopard habitat based on environmental predictors. Annual Mean Temperature (Bio 1) was the most influential variable, contributing 49.3%, followed by elevation (34.4%), precipitation seasonality (Bio 15, 7.9%), mean diurnal range (Bio 2, 4.4%), and precipitation of warmest quarter (Bio 18, 1.5%) (Table 4). Variables such as slope and land cover had minimal influence.

**TABLE 4:** Percentage contribution of variables in building of MaxEnt model.

Variable	Description	Contribution %
Bio 1	Annual Mean Temperature	49.3
elev	Elevation	34.4
Bio 15	Precipitation Seasonality (Coefficient of Variation)	7.9
Bio 2	Mean Diurnal Range (Mean of monthly (max temp–min temp))	4.4
Bio 18	Precipitation of Warmest Quarter	1.5
Soil	Soil Type	0.9
Bio 7	Temperature Annual Range (BIO5‐BIO6)	0.9
Land‐cover	Land Cover Type	0.6
Slope	Slope	0

**FIGURE 4:**
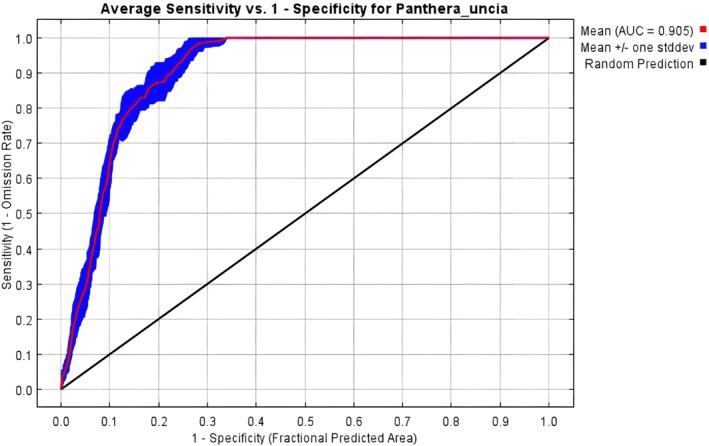
Receiver operating characteristic (ROC) for *Panthera uncia*.

Model evaluation using the Area Under the Curve (AUC) and True Skill Statistic (TSS) metrics demonstrated high predictive performance, with an average AUC of 0.905 (Figure 4) and a mean TSS of 0.74 (Table 5), indicating excellent discriminatory ability and robustness of the model.

**TABLE 5:** Average TSS of the result of the MaxEnt model with 10‐times replication.

Models	Threshold	TSS
Panthera_uncia_0	0.4	0.82
Panthera_uncia_1	0.31	0.72
Panthera_uncia_2	0.3	0.75
Panthera_uncia_3	0.28	0.74
Panthera_uncia_4	0.2	0.70
Panthera_uncia_5	0.3	0.74
Panthera_uncia_6	0.46	0.75
Panthera_uncia_7	0.24	0.73
Panthera_uncia_8	0.41	0.71
Panthera_uncia_9	0.27	0.74
Mean	0.317	0.74

**FIGURE 5:**
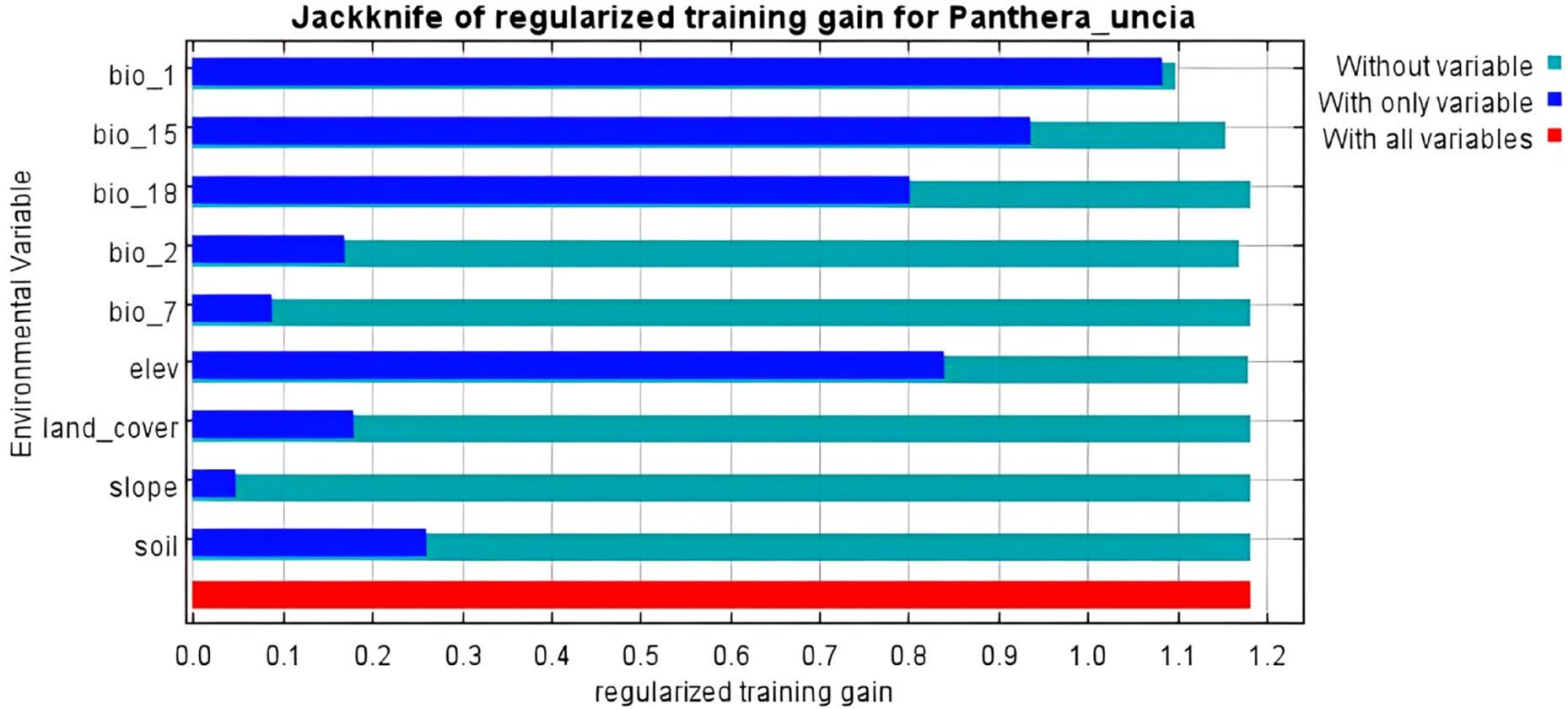
Jackknife of AUC for *Panthera uncia*.

The jackknife analysis reveals how each variable uniquely contributes to the accuracy of the model. The Jackknife analysis revealed that Bio 1 (Annual Mean Temperature) contributed most to the model when variables were tested individually, with Bio 15 (Precipitation Seasonality), elevation, and Bio 18 (Precipitation of Warmest Quarter) ranking next in importance (Figure 5). However, the model's performance declined most when the Bio 1 (Annual Mean Temperature) variable was excluded, indicating that Bio 1 actually provided the most valuable predictive information for the model.

**FIGURE 6:**
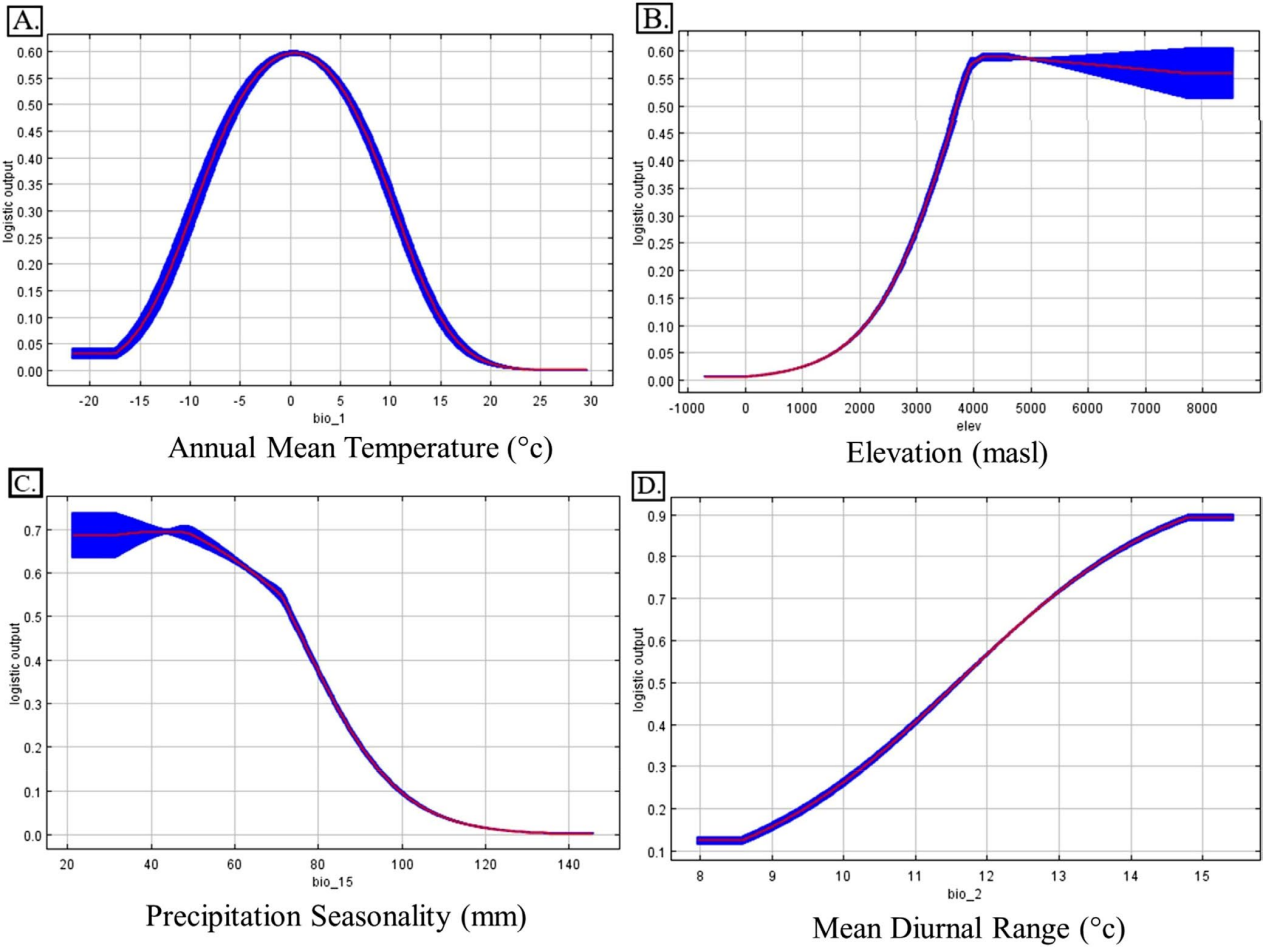
Response curves of major variables influencing the habitat distribution of Snow Leopard in Nepal: (A) Annual Mean Temperature (Bio 1), (B) Elevation, (C) Precipitation Seasonality (Bio15), (D) Mean Diurnal Range (Bio 2).

The response curves (Figure 6) revealed that habitat suitability is highest at lower temperatures (between −5°C and 5°C) and an optimal elevation of 4000–4500 masl. Suitability declines at temperatures above 5°C and at elevations above 4500 m, suggesting a narrow ecological niche for *P. uncia*. The habitat suitability decreased with increased precipitation seasonality (Bio 15) and shows a unimodal response to the diurnal temperature range (Bio 2).

### 3.4 Future Habitat Distribution

**FIGURE 7:**
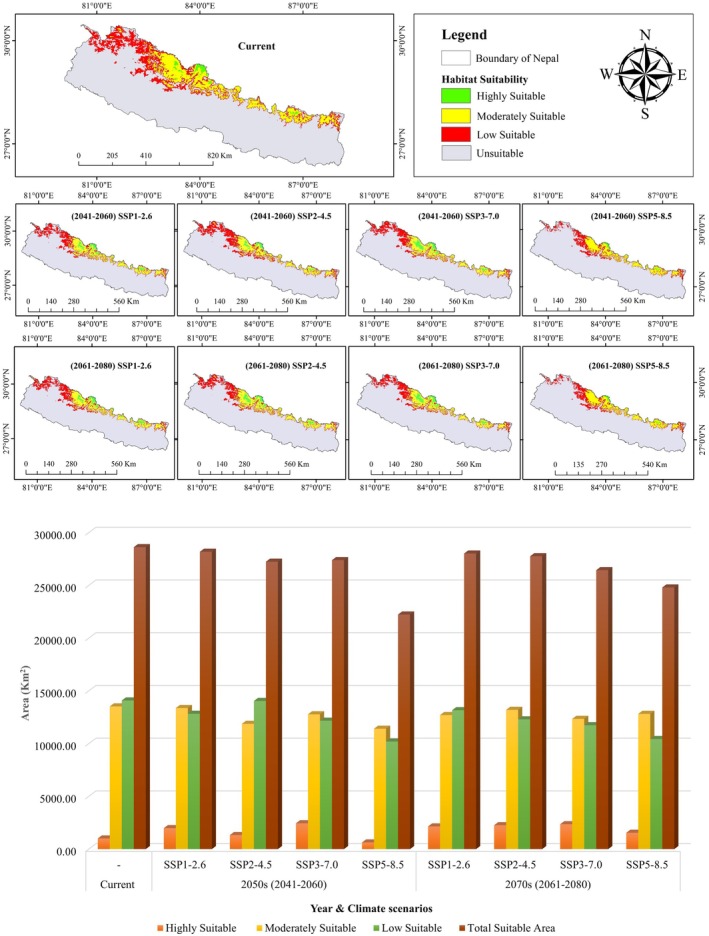
Future distribution of *Panthera uncia* under different climatic scenarios.

Future distribution modeling under four Shared Socioeconomic Pathways (SSPs) (SSP1‐2.6, SSP2‐4.5, SSP3‐7.0, SSP5‐8.5) for the 2050s and 2070s projected a decline in suitable habitat, particularly under high‐emission scenarios (SSP3‐7.0 and SSP5‐8.5) (Figure 7).

Under the high emission scenario (SSP5‐8.5), the total suitable area is expected to shrink to 22,239.23 km^2^ by 2050s and slightly increase to 24,804.95 km^2^ by 2070s, with habitat outside protected areas declining more sharply than inside PAs. While highly suitable habitats show minor fluctuations, moderate and low suitability areas experience significant contraction. These projections emphasize the vulnerability of snow leopard habitats to climate change and highlight the role of PAs in maintaining habitat stability under adverse climatic conditions.

**FIGURE 8:**
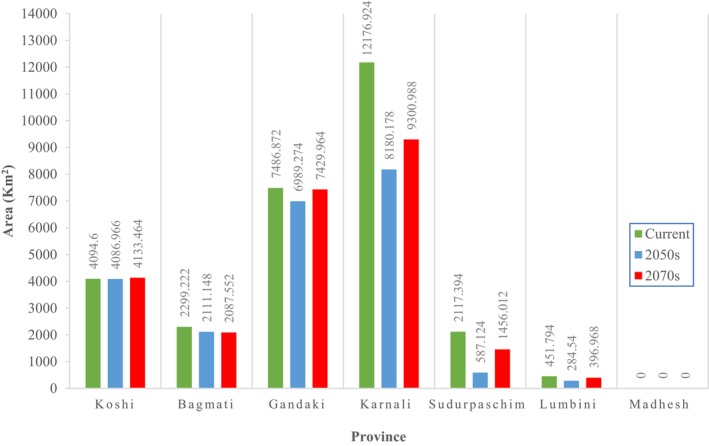
Province‐wise habitat distribution of snow leopard in Nepal under high emission scenario.

Under the high emission scenario (SSP5‐8.5), province‐wise habitat distribution reveals that Karnali and Sudurpaschim provinces experience significant fluctuations in habitat suitability area, while Koshi, Bagmati, and Gandaki provinces show relatively stable conditions (Figure 8).

## 4 Discussion

### 4.1 Habitat Suitability and Comparison With Previous Studies

In Nepal, snow leopard habitat is predominantly distributed across the high Himalayas, exhibiting a spatial pattern consistent with rugged, alpine terrain. Recent Nepalese studies (Dhami et al. 2024; Khanal et al. 2024; Koju et al. 2024; Malla et al. 2023) corroborate our findings, emphasizing the need for region‐specific adaptive management to sustain snow leopard populations under rapid environmental change. Our study estimates that approximately 28,626.81 km^2^ (19.41%) of Nepal's land area is currently suitable for snow leopard, exceeding predictions by Aryal et al. (2016) (22,625.34 km^2^), and Forrest et al. (2012) (20,000 km^2^), but slightly below GSLEP (2013) projection of 30,000 km^2^. These differences likely arise from improvements in species occurrence datasets, higher‐resolution environmental layers, and advancements in the MaxEnt modeling algorithm.

Studies in other regions corroborate climate‐driven habitat reconfigurations. In Qinghai, China, nearly one quarter of the current suitable habitat would be shrunk by the 2050s, from 302,821 km^2^ to 228,997 km^2^, likely due to warming‐induced elevation shifts (Cong et al. 2025; Li et al. 2021). Similarly, the study from Mongolia indicates that the occupancy of snow leopard is concentrated in rugged, arid, mid‐elevation terrain analogous to Nepal's high suitability region suggesting the species' sensitivity to human pressure and climate‐mediated habitat compression (Farrington and Li 2024; Rosenbaum et al. 2023).

Within Nepal, 57.47% (16,451.27 km^2^) of suitable habitat lies within the protected area network, representing a notable increase from the 14,927 km^2^ (Aryal et al. 2016). This suggests improved alignment between conservation landscapes and core snow leopard habitats. The Annapurna Conservation Area (5019 km^2^) and Shey‐Phoksundo National Park (3968 km^2^) emerge as critical refugia, supporting previous findings by Aryal et al. (2016) and Rana (2019). Interestingly, our model identifies Manaslu Conservation Area (1140 km^2^) as more suitable than Kanchenjunga Conservation Area (1061 km^2^), reflecting regional variability in habitat quality and species occurrence. Based on our model, habitat suitability for the snow leopard emphasizes the importance of habitat connectivity and transboundary cooperation in Nepal. In the Himalayas, the shrinkage and shift of habitats are mostly due to climate change, and these are the main factors for the integration of future climatic scenarios. The pinpoint areas that are critical to habitat fragmentation threatening snow leopards are analyzed with spatial connectivity (Li et al. 2020).

Despite these advancements, limitations remain. Reliance on GBIF occurrence data may introduce spatial bias, particularly in remote western regions. Additionally, static prey distribution assumptions do not account for climate‐induced shifts in prey populations, which could indirectly affect habitat suitability. Integrating dynamic prey datasets and fine‐scale field surveys would enhance model accuracy in future studies. Based on the environmental factors such as temperature and elevation, Bai et al. (2018) reported the best model under the mean AUC of 0.921 in the Qomolangma National Nature Reserve, Tibet for snow leopard habitat prediction. Likewise, a study in the high Himalayas by Watts et al. (2019) showed high accuracy and reliability under AUC 0.909 in modeling snow leopard habitat.

### 4.2 Influence of Environmental Variables and Future Uncertainties

MaxEnt modeling identified annual mean temperature (Bio1, 49.3%) and elevation (34.4%) as the strongest predictors of snow leopard habitat, followed by precipitation seasonality (Bio15, 7.9%) and mean diurnal range (Bio2, 4.4%). These results align with global studies emphasizing thermal and precipitation variables in defining the species' ecological niche (Aryal et al. 2016; Ismaili et al. 2024; Shen et al. 2021). Elevation plays a dual role as mid‐altitude zones (3000–5500 m) provide refuge from anthropogenic pressures and offer optimal thermal conditions. In contrast, extreme elevations are less suitable due to harsh climates, sparse vegetation, and limited prey (Bai et al. 2018; Islam et al. 2023). Conversely, lower altitudes are increasingly fragmented by human land use, further restricting viable habitat (Xiao et al. 2022).

The Best model predictions were shown by the most influential factor, Annual Mean Temperature. Such factors represent the strongest control over habitat suitability. In the same way, Precipitation Seasonality and Precipitation of the Warmest Quarter also showed notable contributions to the accuracy of our model. For species distribution, the major drivers, climate variability and topographic conditions, are combinedly monitored by these Bio 15 and Bio 18. The vegetation dynamics and food availability are also dependent on the precipitation patterns of the area (Gao et al. 2023).

Projections under SSP scenarios reveal significant uncertainties, particularly for high‐emission pathways (SSP5‐8.5), where habitat loss variability (±12%) exceeds that of low‐emission scenarios. Precipitation seasonality (Bio15), though secondary to temperature, disproportionately influences model uncertainty, suggesting erratic rainfall patterns may disrupt prey ecology and habitat stability in ways not fully captured by current variables. These findings highlight the need to prioritize climate‐resilient conservation strategies, particularly in western Nepal's non‐PA habitats, where anthropogenic and climatic pressures converge.

### 4.3 Land Use and Anthropogenic Pressures

Land use and land cover (LULC) patterns critically influence habitat fragmentation and landscape connectivity. Consistent with prior studies, snow leopards prefer mosaic alpine habitats, including rocky terrain, snowfields, and meadows (Lamchin et al. 2022). However, the rapid expansion of agriculture, settlement, and transportation infrastructure continues to degrade and fragment these habitats (Mengist et al. 2021). Forest fragmentation, urban encroachment, and unregulated livestock grazing reduce movement corridors essential for gene flow (Khan et al. 2021). These pressures are especially pronounced in lower and mid‐elevation zones, where human encroachment is greatest.

Snow leopard—Human conflict is another major concern. Increased proximity of human activities to habitats has escalated incidences of livestock predation, often resulting in retaliatory killings and weakening support for conservation (Malla et al. 2023; Watts et al. 2019; Zahler and Victurine 2024). Addressing these socio‐ecological challenges requires integrated conservation approaches that balance habitat protection with sustainable livelihoods.

### 4.4 Climate Change and Future Habitat Dynamics

Projected habitat distributions under different Shared Socioeconomic Pathways (SSP) scenarios indicate a substantial decline in suitable habitats, particularly in western Nepal. These trends align with projections, which are consistent with Chapagain et al. (2021), who predicted that climate change would lead to warmer, drier winters and increasingly erratic precipitation patterns in the region. Under high‐emission scenarios (SSP3‐7.0 and SSP5‐8.5), lower elevation habitats are expected to become inhospitable, forcing upward shifts. The impacts of rising temperature and shifting treeline resulted in the fluctuation of the habitat of the snow leopard in the 2050s and 2070s under high‐emission SSP scenarios (Cong et al. 2025; Forrest et al. 2012). In the southern range, the result of rapid warming has caused permafrost thaw, glacier retreat, and shifting of vegetation that has huge impacts on the fragmentation of alpine grasslands, reducing the habitat of the snow leopard (Farrington and Li 2024). The sharper decline outside the PAs aligns with the study that the protected areas (PAs) act as critical refuges that maintain habitat stability (Lham et al. 2021). PAs provide critical habitat continuity for the snow leopard in such a rapidly changing climate (e Hani et al. 2024). Our model indicates that areas currently suitable at lower elevations will become inhospitable, forcing the snow leopard to shift their range upward.

This “elevation‐driven range shifts” mirrors broader patterns observed in Himalayan fauna in response to global warming (Freeman et al. 2018). While low‐emission scenarios may maintain or slightly increase current habitat ranges (Cong et al. 2025), moderate and high‐emission pathways project consistent habitat contraction. Forrest et al. (2012) estimated that up to 30% of the current snow leopard habitat may be lost by 2070 under worst‐case scenarios.

The Himalayas are warming nearly twice as fast as the global average. This rapid change threatens the cold‐adapted alpine ecosystems on which snow leopards depend (Shrestha and Bawa 2014). As these ecosystems contract, suitable habitat becomes increasingly fragmented and isolated, intensifying genetic and demographic risks. This “escalator to extinction” phenomenon reflects the limited vertical migration space available in steep mountain terrains.

Climate‐induced habitat shifts also impact the snow leopard's prey species, including the blue sheep ( *Pseudois nayaur* ) and Himalayan tahr ( *Hemitragus jemlahicus* ), whose distribution and abundance may be altered by thermal changes, leading to potential food scarcity and increased human‐wildlife conflict (Islam et al. 2023). Glacial retreat, permafrost degradation, and loss of alpine wetlands further reduce habitat quality (Aryal et al. 2016; Farrington and Li 2024). Due to the rising temperature and altered rainfall patterns in Karnali and Sudhurpaschim, the contraction of moderate and low suitability habitats was observed (Kazmi et al. 2021).

The spatial heterogeneity in snow leopard habitat across Nepal reflects strong climatic and physiographic gradients. Western Nepal supports more extensive and climatically stable habitats that may serve as future refugia, whereas the central and eastern landscapes are more fragmented due to steeper terrain and greater human pressure. Potential habitat linkages are evident between Annapurna–Manaslu–Langtang–Gaurishankar regions, forming an east–west connectivity axis, while gaps between Shey‐Phoksundo–Api Nampa in the west and Makalu‐Barun–Kanchenjunga in the east suggest fragmentation barriers. Maintaining these corridors through transboundary conservation planning could facilitate range shifts and gene flow under a warming climate (Cong et al. 2025; Li et al. 2020; Rosenbaum et al. 2023).

### 4.5 Conservation Implications

Our results highlight the urgent need to include climate adaptation in snow leopard conservation efforts. This can be done by protecting climate refugia, such as high‐altitude valleys and rugged terrain likely to stay suitable under future climate scenarios, and by creating ecological corridors between protected areas to support altitudinal migration and maintain gene flow. Landscape‐level conservation planning should consider snow leopard habitat needs to reduce fragmentation caused by development, grazing, or tourism, using spatial planning tools to determine buffer zones and corridor locations. Community‐based strategies are crucial, including incentive programs, livestock insurance, eco‐tourism benefits, and local participation in monitoring to lower human‐wildlife conflict and promote stewardship (Jackson and Lama 2016). Ultimately, adaptive management based on regular monitoring of populations and habitats will enable strategies to effectively respond to the combined impacts of climate change, human activities, and habitat fragmentation. Together, these focused actions offer a practical framework to sustain healthy snow leopard populations in Nepal amid current and future environmental challenges.

It is important to note that this study focused primarily on climatic variables in species distribution models under future climate scenarios, while non‐climatic factors such as human activities and ecological disturbances were not incorporated. These factors can have significant impacts on habitat suitability. To strengthen conservation planning, future research should integrate anthropogenic influences alongside climate data, providing a more comprehensive understanding of species distribution and habitat dynamics.

## 5 Conclusion

Our findings demonstrate that snow leopard habitats in Nepal are highly sensitive to climatic shifts, with temperature and elevation exerting the strongest influence on their distribution. While Protected Areas currently encompass the majority of suitable habitats, extensive regions outside these networks remain both ecologically important and disproportionately vulnerable to future contraction. The projected habitat losses under high‐emission scenarios emphasize the need to shift from a protected‐area‐centric approach to adaptive, landscape‐scale conservation that promotes connectivity and climate resilience. Strengthening community‐based stewardship, safeguarding potential climate refugia, and fostering transboundary collaboration will be critical for sustaining viable populations. Future research integrating prey dynamics, anthropogenic pressures, and fine‐scale climate processes will further improve conservation planning and ensure the long‐term persistence of snow leopards across Nepal's rapidly changing mountain landscapes.

## Author Contributions

**Meghajan Budha:** conceptualization (equal), data curation (equal), formal analysis (equal), funding acquisition (equal), investigation (equal), methodology (equal), project administration (equal), resources (equal), software (equal), supervision (equal), validation (equal), visualization (equal), writing – original draft (equal), writing – review and editing (equal). **Jharana Karki:** data curation (equal), formal analysis (equal), methodology (equal), software (equal), writing – original draft (equal), writing – review and editing (equal). **Barsha Khadka:** data curation (equal), formal analysis (equal), methodology (equal), software (equal), writing – original draft (equal), writing – review and editing (equal). **Narayan Prasad Koju:** conceptualization (equal), data curation (equal), formal analysis (equal), funding acquisition (equal), investigation (equal), methodology (equal), project administration (equal), resources (equal), software (equal), supervision (equal), validation (equal), visualization (equal), writing – review and editing (equal).

## Conflicts of Interest

The authors declare no conflicts of interest.

## Data Availability

The data associated with this manuscript are available at https://doi.org/10.5061/dryad.brv15dvmn.

## References

[ece372490-bib-0001] Ale, S. B. , P. Yonzon , and K. Thapa . 2007. “Recovery of Snow Leopard *Uncia uncia* in Sagarmatha (Mount Everest) National Park, Nepal.” Oryx 41, no. 1: 89–92. 10.1017/S0030605307001585.

[ece372490-bib-0002] ANCA . 2018. “Habitat Monitoring of the Snow Leopard *Uncia uncia* in Api Nampa Conservation Area Nepal.”

[ece372490-bib-0003] Aryal, A. , S. Gastaur , S. Menzel , T. B. Chhetri , and J. Hopkins . 2010. “Estimation of Blue Sheep Population Parameters in the Dhorpatan Hunting Reserve, Nepal.” International Journal of Biodiversity and Conservation 2, no. 3: 51–55.

[ece372490-bib-0004] Aryal, A. , U. B. Shrestha , W. Ji , et al. 2016. “Predicting the Distributions of Predator (Snow Leopard) and Prey (Blue Sheep) Under Climate Change in the Himalaya.” Ecology and Evolution 6, no. 12: 4065–4075. 10.1002/ece3.2196. 27516864 PMC4875782

[ece372490-bib-0005] Bai, D.‐F. , P.‐J. Chen , L. Atzeni , L. Cering , Q. Li , and K. Shi . 2018. “Assessment of Habitat Suitability of the Snow Leopard ( *Panthera uncia* ) in Qomolangma National Nature Reserve Based on MaxEnt Modeling.” Zoological Research 39, no. 6: 373–386. 29872029 10.24272/j.issn.2095-8137.2018.057 PMC6085764

[ece372490-bib-0006] Baral, J. 1986. “Nepal: Its Land and Its Uses.” In Land and Its Uses—Actual and Potential: An Environmental Appraisal, 523–534. Springer.

[ece372490-bib-0007] Baral, K. , B. Adhikari , S. Bhandari , et al. 2023. “Impact of Climate Change on Distribution of Common Leopard ( *Panthera pardus* ) and Its Implication on Conservation and Conflict in Nepal.” Heliyon 9, no. 1: e12807. 10.1016/j.heliyon.2023.e12807. 36660456 PMC9843263

[ece372490-bib-0008] Beery, S. , E. Cole , J. Parker , P. Perona , and K. Winner . 2021. “Species Distribution Modeling for Machine Learning Practitioners: A review. Proceedings of the 4th ACM SIGCAS Conference on Computing and Sustainable Societies.”

[ece372490-bib-0009] Bhattacharjee, A. , J. D. Anadón , D. J. Lohman , et al. 2017. “The Impact of Climate Change on Biodiversity in Nepal: Current Knowledge, Lacunae, and Opportunities.” Climate 5, no. 4: 80. 10.3390/cli5040080.

[ece372490-bib-0010] Bhuju, U. R. , P. R. Shakya , T. B. Basnet , and S. Shrestha . 2007. “Nepal Biodiversity Resource Book: Protected Areas, Ramsar Sites, and World Heritage Sites. Environmental Science.”

[ece372490-bib-0011] Boria, R. A. , L. E. Olson , S. M. Goodman , and R. P. Anderson . 2014. “Spatial Filtering to Reduce Sampling Bias Can Improve the Performance of Ecological Niche Models.” Ecological Modelling 275: 73–77. 10.1016/j.ecolmodel.2013.12.012.

[ece372490-bib-0012] Brown, J. L. 2014. “SDM Toolbox: A Python‐Based GIS Toolkit for Landscape Genetic, Biogeographic and Species Distribution Model Analyses.” Methods in Ecology and Evolution 5, no. 7: 694–700. 10.1111/2041-210X.12200. PMC5721907 29230356

[ece372490-bib-0013] Buzzard, P. J. , X. Li , and W. V. Bleisch . 2017. “The Status of Snow Leopards Panthera Uncia, and High Altitude Use by Common Leopards *P. pardus*, in North‐West Yunnan, China.” Oryx 51, no. 4: 587–589.

[ece372490-bib-0014] Byers, A. C. , E. A. Byers , and D. Thapa . 2014. “Conservation and Restoration of Alpine Ecosystems in the Upper Barun Valley, Makalu‐Barun National Park, Nepal. Final Report Submitted to National Geographic Society.”

[ece372490-bib-0015] Chapagain, D. , S. Dhaubanjar , and L. Bharati . 2021. “Unpacking Future Climate Extremes and Their Sectoral Implications in Western Nepal.” Climatic Change 168: 8. 10.1007/s10584-021-03216-8.

[ece372490-bib-0016] Chen, L. , C. Jiang , X. Zhang , et al. 2022. “Prediction of the Potential Distribution of the Predatory Mite *Neoseiulus californicus* (McGregor) in China Under Current and Future Climate Scenarios.” Scientific Reports 12, no. 1: 11807. 10.1038/s41598-022-15308-1. 35821252 PMC9276784

[ece372490-bib-0017] Chen, P. , Y. Gao , J. Wang , et al. 2017. “Status and Conservation of the Endangered Snow Leopard *Panthera uncia* in Qomolangma National Nature Reserve, Tibet.” Oryx 51, no. 4: 590–593.

[ece372490-bib-0018] Chetri, M. , M. Odden , and P. Wegge . 2017. “Snow Leopard and Himalayan Wolf: Food Habits and Prey Selection in the Central Himalayas, Nepal.” PLoS One 12, no. 2: e0170549. 28178279 10.1371/journal.pone.0170549 PMC5298268

[ece372490-bib-0019] Cong, W. , J. Li , Y. Zhang , et al. 2025. “Snow Leopard Habitat Vulnerability Assessment Under Climate Change and Connectivity Corridor in Xinjiang Uygur Autonomous Region, China.” Scientific Reports 15, no. 1: 14583. 10.1038/s41598-025-98909-w. 40281209 PMC12032215

[ece372490-bib-0020] Dhami, B. , N. B. Chhetri , B. Neupane , et al. 2024. “Predicting the Current Habitat Refugia of Himalayan Musk Deer (*Moschus chrysogaster*) Across Nepal.” Ecology and Evolution 14, no. 2: e10949. 10.1002/ece3.10949. 38371859 PMC10870248

[ece372490-bib-0021] DNPWC . 2017. “Snow Leopard Conservation Action Plan (2017–2021). Department of National Parks and Wildlife Conservation.”

[ece372490-bib-0022] DNPWC, & DoFSC . 2024. “Snow Leopard Conservation Action Plan for Nepal (2024–2030). Department of National Parks and Wildlife Conservation and Department of Forests and Soil Conservation.”

[ece372490-bib-0023] DNPWC, & DoFSC . 2025. “Status of Snow Population in Nepal. Department of National Parks and Wildlife Conservation and Department of Forests and Soil Conservation: Ministry of Forests and Environment.”

[ece372490-bib-0024] e Hani, U. , S. M. Haq , R. Shabbir , et al. 2024. “Geospatial Assessment of Climate and Human Pressure on Snow Leopard Habitat in the Trans‐Himalayan Region of Pakistan.” Global Ecology and Conservation 53: e03024. 10.1016/j.gecco.2024.e03024.

[ece372490-bib-0025] Estes, J. A. , J. Terborgh , J. S. Brashares , et al. 2011. “Trophic Downgrading of Planet Earth.” Science 333, no. 6040: 301–306. 10.1126/science.1205106. 21764740

[ece372490-bib-0026] Farrington, J. D. , and J. Li . 2024. “Climate Change Impacts on Snow Leopard Range.” In Snow Leopards, 81–93. Elsevier.

[ece372490-bib-0027] Feng, X. , J. Li , H. Liu , and J. Zhang . 2022. “Impacts of Global Warming on the Snow Leopard. 249–253.” 10.5220/0011198000003443.

[ece372490-bib-0028] Fick, S. E. , and R. J. Hijmans . 2017. “WorldClim 2: New 1‐Km Spatial Resolution Climate Surfaces for Global Land Areas.” International Journal of Climatology 37, no. 12: 4302–4315. 10.1002/joc.5086.

[ece372490-bib-0029] Filla, M. , R. P. Lama , T. Filla , et al. 2022. “Patterns of Livestock Depredation by Snow Leopards and Effects of Intervention Strategies: Lessons From the Nepalese Himalaya.” Wildlife Research 49: 719–737.

[ece372490-bib-0030] Forester, B. R. , E. G. DeChaine , and A. G. Bunn . 2013. “Integrating Ensemble Species Distribution Modelling and Statistical Phylogeography to Inform Projections of Climate Change Impacts on Species Distributions.” Diversity and Distributions 19, no. 12: 1480–1495.

[ece372490-bib-0031] Forrest, J. L. , E. Wikramanayake , R. Shrestha , et al. 2012. “Conservation and Climate Change: Assessing the Vulnerability of Snow Leopard Habitat to Treeline Shift in the Himalaya.” Biological Conservation 150, no. 1: 129–135. 10.1016/j.biocon.2012.03.001.

[ece372490-bib-0032] Freeman, B. G. , M. N. Scholer , V. Ruiz‐Gutierrez , and J. W. Fitzpatrick . 2018. “Climate Change Causes Upslope Shifts and Mountaintop Extirpations in a Tropical Bird Community.” Proceedings of the National Academy of Sciences 115, no. 47: 11982–11987. 10.1073/pnas.1804224115. PMC6255149 30373825

[ece372490-bib-0033] Gama, M. , D. Crespo , M. Dolbeth , and P. M. Anastácio . 2017. “Ensemble Forecasting of *Corbicula fluminea* Worldwide Distribution: Projections of the Impact of Climate Change.” Aquatic Conservation: Marine and Freshwater Ecosystems 27, no. 3: 675–684. 10.1002/aqc.2767.

[ece372490-bib-0034] Gao, X. , S. Bu , and X. Zheng . 2023. “Integrating Species Distribution Models to Estimate the Population Size of Forest Musk Deer (*Moschus berezovskii*) in the Central Qinling Mountains of Shaanxi.” Diversity 15, no. 10: 1071. 10.3390/d15101071.

[ece372490-bib-0035] GBIF . 2025. “GBIF Occurrence.” 10.15468/dl.q8xed8.

[ece372490-bib-0036] GSLEP . 2013. “Global Snow Leopard Ecosystem Protection Program (GSLEP).” Snow Leopard Working Secretariat.

[ece372490-bib-0037] Gurung, S. B. , S. R. Sigdel , and M. B. Rokaya . 2024. “Nepal: An Introduction.” In Flora and Vegetation of Nepal, 1–17. Springer. 10.1007/978-3-031-50702-1_1.

[ece372490-bib-0038] Hacker, C. E. 2021. “Understanding Snow Leopard (*Panthera uncia*) Population Structure, Diet, and Human‐Wildlife Dimensions Using Noninvasive Genetic Approaches Duquesne University.”

[ece372490-bib-0039] Hajian‐Tilaki, K. 2013. “Receiver Operating Characteristic (ROC) Curve Analysis for Medical Diagnostic Test Evaluation.” Caspian Journal of Internal Medicine 4, no. 2: 627. 24009950 PMC3755824

[ece372490-bib-0040] Harris, G. M. , S. E. Sesnie , and D. R. Stewart . 2023. “Climate Change and Ecosystem Shifts in the Southwestern United States.” Scientific Reports 13, no. 1: 19964. 10.1038/s41598-023-46371-x. 37968297 PMC10651835

[ece372490-bib-0041] Ikeda, N. 2004. “Economic Impacts of Livestock Depredation by Snow Leopard *Uncia uncia* in the Kanchenjunga Conservation Area, Nepal Himalaya.” Environmental Conservation 31, no. 4: 322–330. 10.1017/S0376892904001778.

[ece372490-bib-0042] IPCC . 2023. Climate Change 2023: Synthesis Report. Contribution of Working Groups I, II and III to the Sixth Assessment Report of the Intergovernmental Panel on Climate Change, edited by Core Writing Team , H. Lee , and J. Romero , 184. IPCC. 10.59327/IPCC/AR6-9789291691647.

[ece372490-bib-0043] Islam, M. , M. Sahana , G. Areendran , C. Jamir , K. Raj , and H. Sajjad . 2023. “Prediction of Potential Habitat Suitability of Snow Leopard ( *Panthera uncia* ) and Blue Sheep ( *Pseudois nayaur* ) and Niche Overlap in the Parts of Western Himalayan Region.” Geo: Geography and Environment 10, no. 1: e00121.

[ece372490-bib-0044] Ismaili, R. R. R. , X. Peng , Y. Li , et al. 2024. “Modeling Habitat Suitability of Snow Leopards in Yanchiwan National Reserve, China.” Animals 14, no. 13: 1938. 10.3390/ani14131938. 38998049 PMC11240653

[ece372490-bib-0045] Jackson, R. M. , and W. B. Lama . 2016. “The Role of Mountain Communities in Snow Leopard Conservation.” In Snow Leopards, 139–149. Elsevier.

[ece372490-bib-0046] Jackson, R. M. , C. Mishra , T. M. McCarthy , and S. B. Ale . 2010. “Snow Leopards: Conflict and Conservation.” Biology and Conservation of Wild Felids 2: 417–430.

[ece372490-bib-0047] Janečka, J. , R. Jackson , Z. Yuquang , et al. 2008. “Population Monitoring of Snow Leopards Using Noninvasive Collection of Scat Samples: A Pilot Study.” Animal Conservation 11, no. 5: 401–411. 10.1111/j.1469-1795.2008.00195.x.

[ece372490-bib-0048] Jianhui, G. , L. Yibin , W. Ruifen , Y. Chenxing , F. Jian , and S. Kun . 2023. “MaxEnt Modeling for Predicting Suitable Habitats of Snow Leopard (*Panthera uncia*) in the Mid‐Eastern Tianshan Mountains.” Journal of Resources and Ecology 14, no. 5: 1075–1085. 10.5814/j.issn.1674-764x.2023.05.018.

[ece372490-bib-0049] Jnawali, S. R. , H. S. Baral , S. Lee , et al. 2011. “The Status of Nepal's Mammals: The National Red List Series. Department of National Parks and Wildlife Conservation.”

[ece372490-bib-0050] Karki, A. , and S. Panthi . 2021. “Factors Affecting Livestock Depredation by Snow Leopards ( *Panthera uncia* ) in the Himalayan Region of Nepal.” PeerJ 9: e11575. 10.7717/peerj.11575. 34178460 PMC8214393

[ece372490-bib-0051] Kazmi, F. A. , F. Shafique , M. Hassan , et al. 2021. “Ecological Impacts of Climate Change on the Snow Leopard (*Panthera unica*) in South Asia.” Brazilian Journal of Biology 82: e240219. 10.1590/1519-6984.240219 34105645

[ece372490-bib-0052] Khan, T. U. , A. Mannan , C. E. Hacker , et al. 2021. “Use of GIS and Remote Sensing Data to Understand the Impacts of Land Use/Land Cover Changes (LULCC) on Snow Leopard (*Panthera uncia*) Habitat in Pakistan.” Sustainability 13, no. 7: 3590.

[ece372490-bib-0053] Khanal, G. , C. Mishra , and K. Ramesh Suryawanshi . 2020. “Relative Influence of Wild Prey and Livestock Abundance on Carnivore‐Caused Livestock Predation.” Ecology and Evolution 10, no. 20: 11787–11797. 10.1002/ece3.6815. 33145001 PMC7593152

[ece372490-bib-0054] Khanal, G. , K. B. Shah , R. M. Jackson , and S. Ale . 2024. “Conservation of Snow Leopard in Nepal.” In Snow Leopards, 531–539. Elsevier.

[ece372490-bib-0055] Koju, N. P. , B. Bashyal , B. P. Pandey , S. N. Shah , S. Thami , and W. V. Bleisch . 2021. “First Camera‐Trap Record of the Snow Leopard *Panthera uncia* in Gaurishankar Conservation Area, Nepal.” Oryx 55, no. 2: 173–176. 10.1017/S003060532000006X.

[ece372490-bib-0056] Koju, N. P. , P. Buzzard , A. Shrestha , et al. 2024. “Habitat Overlap and Interspecific Competition Between Snow Leopards and Leopards in the Central Himalayas of Nepal.” Global Ecology and Conservation 52: e02953. 10.1016/j.gecco.2024.e02953.

[ece372490-bib-0057] Koju, N. P. , K. R. Gosai , B. Bashyal , et al. 2023. “Seasonal Prey Abundance and Food Plasticity of the Vulnerable Snow Leopard (*Panthera uncia*) in the Lapchi Valley, Nepal Himalayas.” Animals 13, no. 20: 3182. 10.3390/ani13203182. 37893906 PMC10603713

[ece372490-bib-0058] Lama, R. P. , T. R. Ghale , M. K. Suwal , R. Ranabhat , and G. R. Regmi . 2018. “First Photographic Evidence of Snow Leopard *Panthera uncia* (Mammalia: Carnivora: Felidae) Outside Current Protected Areas Network in Nepal Himalaya.” Journal of Threatened Taxa 10, no. 8: 12086–12090.

[ece372490-bib-0059] Lamchin, M. , W.‐K. Lee , and S. W. Wang . 2022. “Multi‐Temporal Analysis of Past and Future Land‐Cover Changes of the Third Pole.” Land 11, no. 12: 2227.

[ece372490-bib-0060] Lham, D. , G. Cozzi , S. Sommer , et al. 2021. “Modeling Distribution and Habitat Suitability for the Snow Leopard in Bhutan.” Frontiers in Conservation Science 2: 781085. 10.3389/fcosc.2021.781085.

[ece372490-bib-0061] Li, J. , B. V. Weckworth , T. M. McCarthy , et al. 2020. “Defining Priorities for Global Snow Leopard Conservation Landscapes.” Biological Conservation 241: 108387.

[ece372490-bib-0062] Li, J. , Y. Xue , C. E. Hacker , et al. 2021. “Projected Impacts of Climate Change on Snow Leopard Habitat in Qinghai Province, China.” Ecology and Evolution 11, no. 23: 17202–17218. 10.1002/ece3.8358. 34938503 PMC8668752

[ece372490-bib-0063] Li, S. , Z. Wang , Z. Zhu , Y. Tao , and J. Xiang . 2023. “Predicting the Potential Suitable Distribution Area of *Emeia pseudosauteri* in Zhejiang Province Based on the MaxEnt Model.” Scientific Reports 13, no. 1: 1806. 10.1038/s41598-023-29009-w. 36721021 PMC9889780

[ece372490-bib-0064] Liu, C. , P. M. Berry , T. P. Dawson , and R. G. Pearson . 2005. “Selecting Thresholds of Occurrence in the Prediction of Species Distributions.” Ecography 28: 385–393.

[ece372490-bib-0065] Liu, C. , G. Newell , and M. White . 2016. “On the Selection of Thresholds for Predicting Species Occurrence With Presence‐Only Data.” Ecology and Evolution 6, no. 1: 337–348. 10.1002/ece3.1878. 26811797 PMC4716501

[ece372490-bib-0066] Lobo, J. M. , A. Jiménez‐Valverde , and R. Real . 2008. “AUC: A Misleading Measure of the Performance of Predictive Distribution Models.” Global Ecology and Biogeography 17, no. 2: 145–151. 10.1111/j.1466-8238.2007.00358.x.

[ece372490-bib-0067] Lovari, S. , S. Kachel , L. Xueyang , and F. Ferretti . 2024. “Snow Leopard, Common Leopard, and Wolf: Are They Good Neighbors?” In Snow Leopards, 137–147. Elsevier.

[ece372490-bib-0068] Lovari, S. , I. Minder , F. Ferretti , N. Mucci , E. Randi , and B. Pellizzi . 2013. “Common and Snow Leopards Share Prey, but Not Habitats: Competition Avoidance by Large Predators?” Journal of Zoology 291, no. 2: 127–135. 10.1111/jzo.12053.

[ece372490-bib-0069] Luo, Y. , J. Yang , L. Liu , and K. Zhang . 2025. “MaxEnt Modeling and Effects of Climate Change on Shifts in Habitat Suitability for *Sorbus alnifolia* in China.” Plants 14, no. 5: 677. 10.3390/plants14050677. 40094567 PMC11901521

[ece372490-bib-0070] Lyngdoh, S. , S. Shrotriya , S. P. Goyal , H. Clements , M. W. Hayward , and B. Habib . 2014. “Prey Preferences of the Snow Leopard ( *Panthera uncia* ): Regional Diet Specificity Holds Global Significance for Conservation.” PLoS One 9, no. 2: e88349. 10.1371/journal.pone.0088349. 24533080 PMC3922817

[ece372490-bib-0071] Mahmood, T. , A. Younas , F. Akrim , S. Andleeb , A. Hamid , and M. S. Nadeem . 2019. “Range Contraction of Snow Leopard ( *Panthera uncia* ).” PLoS One 14, no. 8: e0218460. 10.1371/JOURNAL.PONE.0218460. 31369579 PMC6675083

[ece372490-bib-0072] Malla, R. , S. Panthi , H. Adhikari , et al. 2023. “Habitat Suitability of Four Threatened Himalayan Species: Asiatic Black Bear, Common Leopard, Musk Deer, and Snow Leopard.” PeerJ 11: e16085. 37780372 10.7717/peerj.16085 PMC10538300

[ece372490-bib-0073] McCarthy, T. , D. Mallon , R. Jackson , P. Zahler , and K. McCarthy . 2017. “ *Panthera uncia* . The IUCN Red List of Threatened Species 2017: e. T22732A50664030. The IUCN Red List of Threatened Species.”

[ece372490-bib-0074] McCarthy, T. , D. Mallon , and P. Zahler . 2024. “What Is a Snow Leopard? Biogeography and Status Overview.” In Biodiversity of World: Conservation From Genes to Landscapes, edited by D. Mallon and T. McCarthy , 31–41. Academic Press. 10.1016/B978-0-323-85775-8.00056-X.

[ece372490-bib-0075] Mengist, W. , T. Soromessa , and G. L. Feyisa . 2021. “Landscape Change Effects on Habitat Quality in a Forest Biosphere Reserve: Implications for the Conservation of Native Habitats.” Journal of Cleaner Production 329: 129778.

[ece372490-bib-0076] MoFSC . 2017. “Snow Leopard and Ecosystem Management Plan (2017–2026).”

[ece372490-bib-0077] Moheb, Z. , T. K. Fuller , and P. I. Zahler . 2023. “Snow Leopard‐Human Conflict as a Conservation Challenge—A Review.” Snow Leopard Reports 1: 11–24. 10.56510/slr.v1.8158.

[ece372490-bib-0078] Nowell, K. 2016. “An Ounce of Prevention: Snow Leopard Crime Revisited.”

[ece372490-bib-0079] Oberosler, V. , S. Tenan , C. Groff , et al. 2022. “First Spatially‐Explicit Density Estimate for a Snow Leopard Population in the Altai Mountains.” Biodiversity and Conservation 31, no. 1: 261–275. 10.1007/S10531-021-02333-1.

[ece372490-bib-0080] Pandey, B. P. , S. Thami , R. Shrestha , N. Subedi , M. K. Chalise , and S. B. Ale . 2021. “Snow Leopards and Prey in Rol‐waffling Valley, Gaurishankar Conservation Area, Nepal.”

[ece372490-bib-0081] Phillips, S. J. , R. P. Anderson , and R. E. Schapire . 2006. “Maximum Entropy Modeling of Species Geographic Distributions.” Ecological Modelling 190, no. 3–4: 231–259. 10.1016/j.ecolmodel.2005.03.026.

[ece372490-bib-0082] Piquet, J. C. , M. López‐Darias , A. van der Marel , M. Nogales , and J. Waterman . 2018. “Unraveling Behavioral and Pace‐Of‐Life Syndromes in a Reduced Parasite and Predation Pressure Context: Personality and Survival of the Barbary Ground Squirrel.” Behavioral Ecology and Sociobiology 72, no. 9: 147. 10.1007/s00265-018-2549-8.

[ece372490-bib-0083] Radosavljevic, A. , and R. P. Anderson . 2014. “Making Better Maxent Models of Species Distributions: Complexity, Overfitting and Evaluation.” Journal of Biogeography 41, no. 4: 629–643. 10.1111/jbi.12227.

[ece372490-bib-0084] Rai, S. K. , G. Rai , K. Hirai , A. Abe , and Y. Ohno . 2001. “The Health System in Nepal—An Introduction.” Environmental Health and Preventive Medicine 6, no. 1: 1–8. 21432230 10.1007/BF02897302 PMC2723647

[ece372490-bib-0085] Rana, D. B. 2019. “Distribution Occupancy, Potential Suitable Habitat and Conservation of Recolonized Wolf in Annapurna Conservation Area, Nepal.”

[ece372490-bib-0086] Rashid, W. , J. Shi , I. Rahim , et al. 2021. “Modelling Potential Distribution of Snow Leopards in Pamir, Northern Pakistan: Implications for Human–Snow Leopard Conflicts.” Sustainability 13, no. 23: 13229. 10.3390/su132313229.

[ece372490-bib-0087] Raymond, C. V. , J. L. McCune , H. Rosner‐Katz , et al. 2020. “Combining Species Distribution Models and Value of Information Analysis for Spatial Allocation of Conservation Resources.” Journal of Applied Ecology 57, no. 4: 819–830.

[ece372490-bib-0088] Rosenbaum, B. , A. D. Poyarkov , B. Munkhtsog , et al. 2023. “Seasonal Space Use and Habitat Selection of GPS Collared Snow Leopards (*Panthera uncia*) in the Mongolian Altai Range.” PLoS One 18, no. 1: e0280011. 36649338 10.1371/journal.pone.0280011 PMC10045553

[ece372490-bib-0089] Sanyal, O. , T. Bashir , M. Rana , and P. Chandan . 2024. “First Photographic Record of the Snow Leopard *Panthera uncia* in Kishtwar High Altitude National Park, Jammu and Kashmir, India.” Oryx 58: 1–5. 10.1017/s0030605323001965.

[ece372490-bib-0090] Shen, T. , H. Yu , and Y.‐Z. Wang . 2021. “Assessing the Impacts of Climate Change and Habitat Suitability on the Distribution and Quality of Medicinal Plant Using Multiple Information Integration: Take Gentiana Rigescens as an Example.” Ecological Indicators 123: 107376.

[ece372490-bib-0091] Shrestha, A. B. , and R. Aryal . 2011. “Climate Change in Nepal and Its Impact on Himalayan Glaciers.” Regional Environmental Change 11: 65–77. 10.1007/s10113-010-0174-9.

[ece372490-bib-0092] Shrestha, A. B. , C. P. Wake , J. E. Dibb , and P. A. Mayewski . 2000. “Precipitation Fluctuations in the Nepal Himalaya and Its Vicinity and Relationship With Some Large Scale Climatological Parameters.” International Journal of Climatology: A Journal of the Royal Meteorological Society 20, no. 3: 317–327.

[ece372490-bib-0093] Shrestha, B. , and P. Kindlmann . 2020. “Implications of Landscape Genetics and Connectivity of Snow Leopard in the Nepalese Himalayas for Its Conservation.” Scientific Reports 10, no. 1: 19853. 10.1038/s41598-020-76912-7. 33199758 PMC7669836

[ece372490-bib-0094] Shrestha, U. B. , and K. S. Bawa . 2014. “Impact of Climate Change on Potential Distribution of Chinese Caterpillar Fungus (*Ophiocordyceps sinensis*) in Nepal Himalaya.” PLoS One 9, no. 9: e106405. 25180515 10.1371/journal.pone.0106405 PMC4152242

[ece372490-bib-0095] Thapa, K. , and S. Rayamajhi . 2023. “Anti‐Predator Strategies of Blue Sheep (Naur) Under Varied Predator Compositions: A Comparison of Snow Leopard‐Inhabited Valleys With and Without Wolves in Nepal.” Wildlife Research 51, no. 1: 23012. 10.1071/WR23012.

[ece372490-bib-0096] Thapa, K. , N. Schmitt , N. M. Pradhan , H. R. Acharya , and S. Rayamajhi . 2021. “No Silver Bullet? Snow Leopard Prey Selection in mt. Kangchenjunga, Nepal.” Ecology and Evolution 11, no. 23: 16413–16425. 10.1002/ece3.8279. 34938445 PMC8668728

[ece372490-bib-0097] Theile, S. 2003. “Fading Footprints: The Killing and Trade of Snow Leopards.”

[ece372490-bib-0098] Timilsina, S. , B. P. Pandey , B. Neupane , et al. 2024. “Winter Diet Pattern of Snow Leopard and Factors Affecting Livestock Depredation in Nubri Valley of Manaslu Conservation Area, Nepal.” Ecologies 6, no. 1: 1. 10.3390/ecologies6010001.

[ece372490-bib-0099] Tiwari, M. P. , B. P. Devkota , R. M. Jackson , B. B. K. Chhetri , and S. Bagale . 2020. “What Factors Predispose Households in Trans‐Himalaya (Central Nepal) to Livestock Predation by Snow Leopards?” Animals 10, no. 11: 2187. 10.3390/ani10112187. 33238383 PMC7700291

[ece372490-bib-0100] Upadhyay, M. 2010. “Relative Abundance, Habitat Preference and Threats of Snow Leopard *Uncia uncia* in Upper Mustang, Nepal Tribhuvan University]. Kathmandu, Nepal.”

[ece372490-bib-0101] Wang, R. , C. Jiang , L. Liu , et al. 2021. “Prediction of the Potential Distribution of the Predatory Mite Neoseiulus Californicus McGregor in China Using MaxEnt.” Global Ecology and Conservation 29: e01733. 10.1016/j.gecco.2021.e01733. PMC9276784 35821252

[ece372490-bib-0102] Warren, D. L. , and S. N. Seifert . 2011. “Ecological Niche Modeling in Maxent: The Importance of Model Complexity and the Performance of Model Selection Criteria.” Ecological Applications 21, no. 2: 335–342. 21563566 10.1890/10-1171.1

[ece372490-bib-0103] Watts, S. M. , T. M. McCarthy , and T. Namgail . 2019. “Modelling Potential Habitat for Snow Leopards (*Panthera uncia*) in Ladakh, India.” PLoS One 14, no. 1: e0211509. 30695083 10.1371/journal.pone.0211509 PMC6350993

[ece372490-bib-0104] Wei, T. , V. Simko , M. Levy , et al. 2024. “R Package ‘Corrplot’: Visualization of a Correlation Matrix.” https://github.com/taiyun/corrplot.

[ece372490-bib-0105] WWF . 2024. “Snow Leopard Population Outside Protected Area of Dolpa unveiled. WWF Nepal.” https://www.wwfnepal.org/?384856%2FSnow‐leopard‐population‐outside‐Protected‐Area‐of‐Dolpa‐unveiled.

[ece372490-bib-0106] Xiao, L. , F. Hua , J. Knops , et al. 2022. “Spatial Separation of Prey From Livestock Facilitates Coexistence of a Specialized Large Carnivore With Human Land Use.” Animal Conservation 25, no. 5: 638–647.

[ece372490-bib-0107] Xu, K. , W. Xiao , D. Hu , et al. 2024. “Effects of Livestock Grazing on Spatiotemporal Interactions Between Snow Leopards and Ungulate Prey.” Integrative Zoology 20: 1012–1027. 10.1111/1749-4877.12935. 39687974

[ece372490-bib-0108] Young, N. , L. Carter , and P. Evangelista . 2011. “A MaxEnt Model v3. 3.3 e Tutorial (ArcGIS v10). Natural Resource Ecology Laboratory, Colorado State University and the National Institute of Invasive Species Science.”

[ece372490-bib-0109] Zahler, P. , and R. Victurine . 2024. “Linear Infrastructure and Snow Leopard Conservation.” In Snow Leopards, 123–128. Elsevier.

[ece372490-bib-0110] Zahoor, B. , X. Liu , P. Wu , et al. 2021. “Activity Pattern Study of Asiatic Black Bear (*Ursus thibetanus*) in the Qinling Mountains, China, by Using Infrared Camera Traps.” Environmental Science and Pollution Research 28: 25179–25186. 33447985 10.1007/s11356-020-12325-3

[ece372490-bib-0111] Zurell, D. , J. Franklin , C. König , et al. 2020. “A Standard Protocol for Reporting Species Distribution Models.” Ecography 43, no. 9: 1261–1277. 10.1111/ecog.04960.

[ece372490-bib-0112] Zurell, D. , C. König , A. K. Malchow , et al. 2022. “Spatially Explicit Models for Decision‐Making in Animal Conservation and Restoration.” Ecography 2022, no. 4: 5787.

